# Frequent in Dementia, Deadliest Without It: Delirium and Mortality in Hospitalized Older Adults

**DOI:** 10.1093/geroni/igaf122.3775

**Published:** 2025-12-31

**Authors:** Mfon Umoh, Thiago Junqueira Avelino-Silva, Marlon Juliano Romero Aliberti, Flavia Barreto Garcez, Esther Oh

**Affiliations:** Johns Hopkins University School of Medicine, Baltimore, Maryland, United States; University of California, San Francisco, University of California, San Francisco, California, United States; University of Sao Paulo Medical School, Baltimore, Maryland, United States; University of Sao Paulo Medical School, Sao Paulo, Sao Paulo, Brazil; Johns Hopkins School of Medicine, Johns Hopkins School of Medicine, Maryland, United States

## Abstract

Dementia increases vulnerability to delirium and adverse outcomes, yet the prognostic role of delirium across cognitive status is uncertain. Using data from the CHANGE Study, a prospective cohort of adults aged ≥65 years admitted to 43 hospitals in five countries (Brazil-Angola-Chile-Columbia-Portugal), we examined the association between delirium and 90-day mortality and the modifying effect of cognitive status. Delirium (prevalent and incident) was assessed using the Confusion Assessment Method (CAM), and baseline cognitive status with the Clinical Dementia Rating (CDR). Multi-level mixed effects survival models, adjusted for sociodemographic, clinical, and hospital-related factors, were used to examine the association between delirium and mortality, with stratified analyses by CDR to test interaction. Among 2,556 patients (mean age=79±9, 56% female), 37% experienced delirium during hospitalization. However, delirium occurrence increased significantly with worsening baseline cognitive status, affecting 126/765 (16%) patients with normal cognition (CDR=0), 266/976 (27%) with mild cognitive impairment (CDR=0.5), 208/350 (59%) with mild dementia (CDR=1), and 357/465 (77%) with moderate/severe dementia (CDR=2-3), p < 0.001. After adjustment, delirium was associated with increased 90-day mortality (HR = 3.43, 95%CI=2.81-4.18). The largest mortality difference between patients with and without delirium was observed in those with normal cognition (63% vs. 18%; HR = 4.36, 95%CI=3.11-6.11), and the smallest difference in those with moderate/severe dementia (41% vs. 26%; HR = 2.22, 95%CI=1.34-3.66), with a p-value for interaction=0.04. While delirium was more common among patients with dementia, its prognostic impact on mortality was strongest in patients with normal cognition at baseline. Hospital-wide strategies to urgently detect, prevent, and manage delirium are critical for all older patients.

